# Maternal mirror syndrome with foetal hydrops due to isoimunization by anti‐KPa antibodies: A case report and narrative literature review

**DOI:** 10.1002/ccr3.5484

**Published:** 2022-02-16

**Authors:** Juan Pina Moreno, Virginia Ortega Abad, Ana Perez Corral, Santiago Garcia‐Tizon Larroca

**Affiliations:** ^1^ Gregorio Marañon Mother and Child Hospital Madrid Spain; ^2^ Hematology Hospital General Universitario Gregorio Marañon Madrid Spain

**Keywords:** fetal anemia, hydrops fetalis, immune hydrops, isoimmunization, mirror syndrome

## Abstract

We present a rare case of mirror syndrome due to anti‐Kpa antibodies, which can be difficult to identify with routine screening tests.

## Graphical abstract

The association of fetal hydrops with maternal edema should prompt the suspicion of mirror syndrome. In the consideration of fetal hydrops, it is important to include immunohematological studies that rule out the presence of rare antibodies.

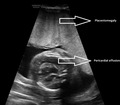

## 1 INTRODUCTION

Mirror syndrome was first described in 1892 by Ballantyne. It presents a clinical picture of maternal edema associated with fetal hydrops and placentomegaly, which must both be present. It is a rare entity, and in many cases, it is underdiagnosed in routine medical practice. Its actual incidence is unknown, and descriptions of this pathology are scarce in the literature. Proof of this is a recent literature review with a sample of only 113 cases reported between 1956 and 2016.^1^ Maternal edema, which is the most characteristic sign and symptom, is clinically diagnosed by the presence of fovea after applying digital pressure for five seconds.^2^ Hydrops fetalis is the pathological accumulation of excess fluid in two or more fetal compartments.^3^ Placentomegaly is defined as a placental thickness greater than 4 cm in the second trimester or greater than 6 cm in the third trimester, according to the published literature.^4^

Regarding the main causes of mirror syndrome, the most frequent is fetal hydrops, which can have an immunological or nonimmunological etiology and can also be due to viral infections or fetal malformations. Other symptoms that are commonly present in pregnant women with this syndrome are arterial hypertension, mild anemia due to hemodilution, proteins in the urine, and elevated liver enzymes. In a high proportion of cases, perinatal death can occur as an adverse event of pregnancy.^5^

Erythrocyte alloimmunization during pregnancy is caused by the presence of maternal antibodies against antigens present in fetal red blood cells. The destruction of fetal erythrocytes, together with liver damage and endothelial injury resulting from hypoxia, is a sufficient pathophysiological mechanism for the development of hydrops in the fetus. This immune mechanism responsible for hydrops occurs in approximately 15% of cases detected prenatally by ultrasound examination.^6^, ^7^

Nonimmune hydrops can have multiple causes, including viral infections, genetic syndromes, fetal heart rhythm disorders, and malformations. At present, it is much more common than cases with immune causes due to the establishment of programs for the prevention of isoimmunization during pregnancy.^3^, ^8^ In particular, the incidence of immune hydrops has decreased since 1970s due to the use of anti‐D immunoglobulin and the establishment of screening programs at the population level.^9^ Most clinically significant maternal erythrocyte alloantibodies are adequately detected with routine screening during pregnancy with an indirect Coomb's test; however, there are cases of maternal‐fetal isoimmunization due to erythrocyte antibodies against very low frequency and atypical erythrocyte antigens. These may not be detected with routine tests and can lead to severe neonatal hemolytic disease.^10^

## 2 CASE HISTORY/EXAMINATION

Our case is a 33‐year‐old woman who was 25 weeks pregnant and had adequate gestational control until she came to the emergency room due to marked edema of the lower limbs. The patient did not report any relevant medical history, and her pregnancy proceeded normally throughout the follow‐up. There were no complications during her previous pregnancy with the same partner.

Upon arrival at our center, the physical examination showed repeated blood pressure measurements of approximately 140/90 and intense edema in both lower limbs with pitting reaching the knee. The patient also had marked edema of the face and neck.

## 3 INVESTIGATIONS AND TREATMENT

The patient underwent various studies described below.

A blood test showed mild anemia with hemodilution (hemoglobin 11.8 g/dl and hematocrit 33.8%), significant proteinuria (urine protein/creatinine index of 0.47 mg/mg), and transaminasemia (ALT 54 U/L. AST 37 U/L) as the most remarkable data. The cardiotocographic record showed a normal preterm fetal pattern and regular uterine dynamics. The abdominal ultrasound examination revealed fetal hydrops with ascites and pericardial effusion, and suspicion of placentomegaly. The patient was admitted to the hospital for maternal and fetal hydrops and monitoring of uterine dynamics.

The next day, a fetal morphological ultrasound was performed that confirmed the presence of ascites with displacement of the intestinal loops to the pelvis and placentomegaly, with a thickness of 80 mm. In addition, pericardial effusion marked by mild cardiomegaly and significant generalized fetal subcutaneous edema was visualized. No other morphological alterations were found, and the complete Doppler study did not demonstrate other hemodynamic alterations. The maximum velocity of the middle cerebral artery was normal in several repeated measurements (36 cm/s, MoM 1.1), and fetal anemia could not be diagnosed at the time of the examination.

The serological study was negative for the presence of maternal‐fetal infections associated with fetal hydrops (parvovirus B19, toxoplasma, rubella, syphilis, EBV, VH6, VZV, and HIV). An indirect Coomb's study with the usual panel was negative.

**FIGURE 1:**
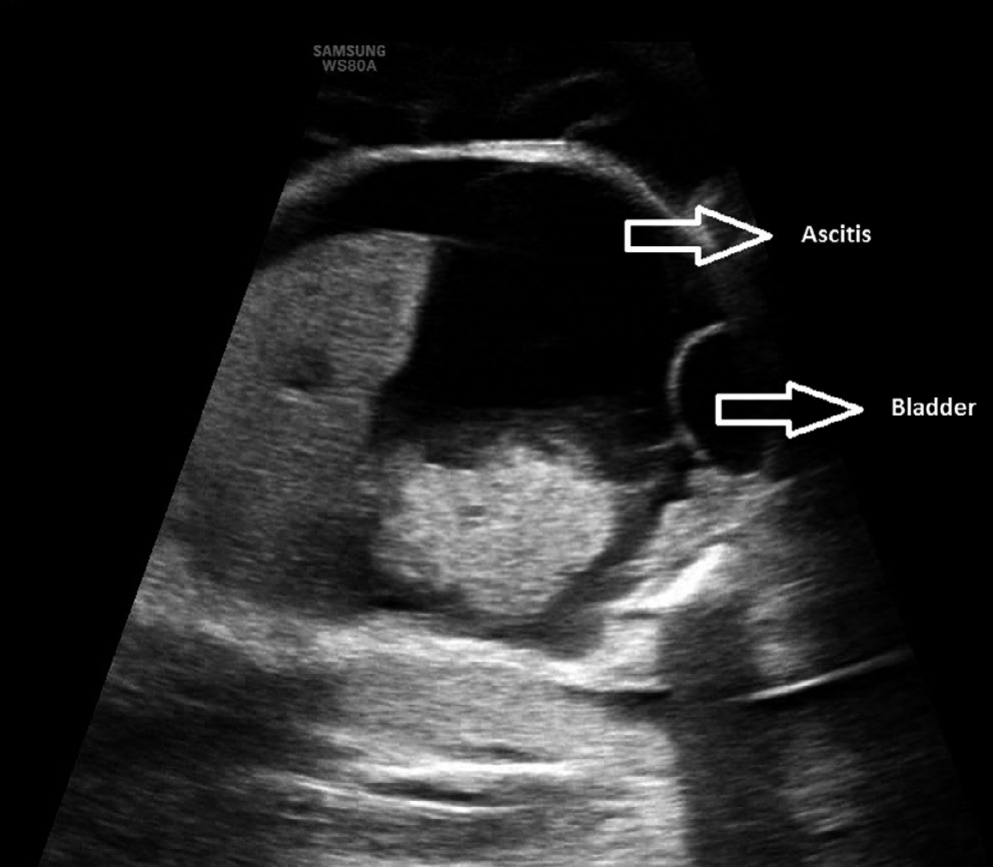
Fetal ascites

**FIGURE 2:**
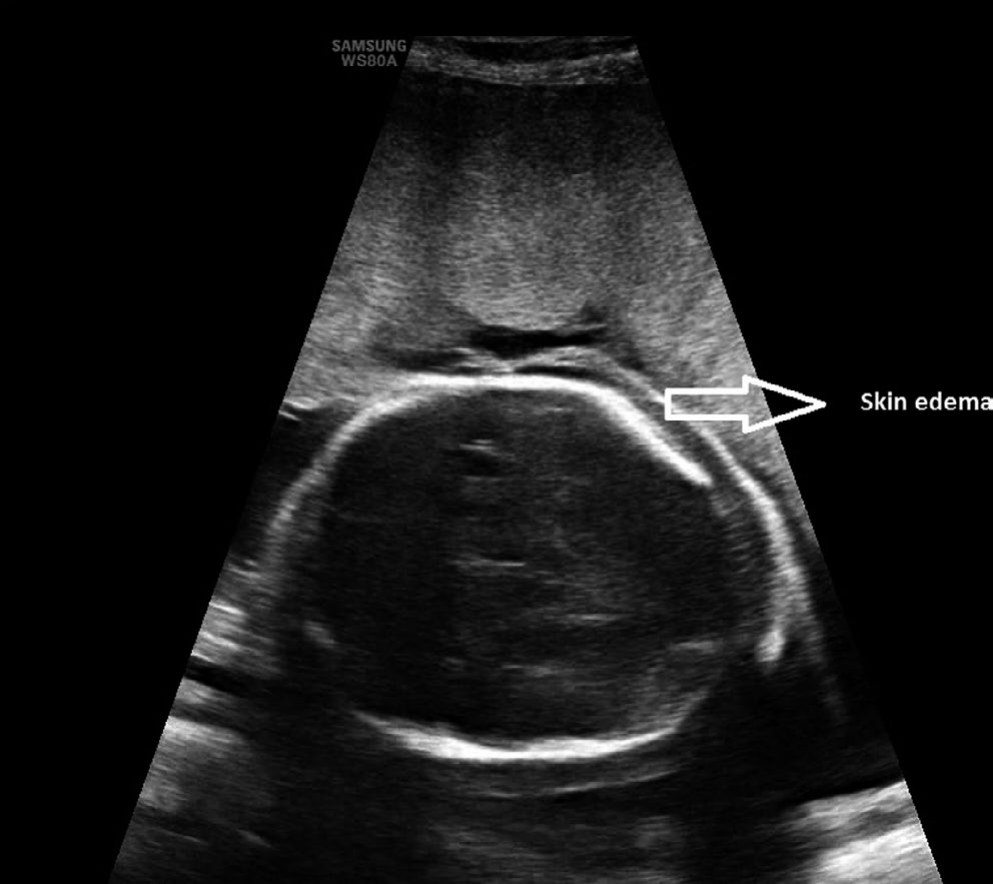
Fetal skin edema

**FIGURE 3:**
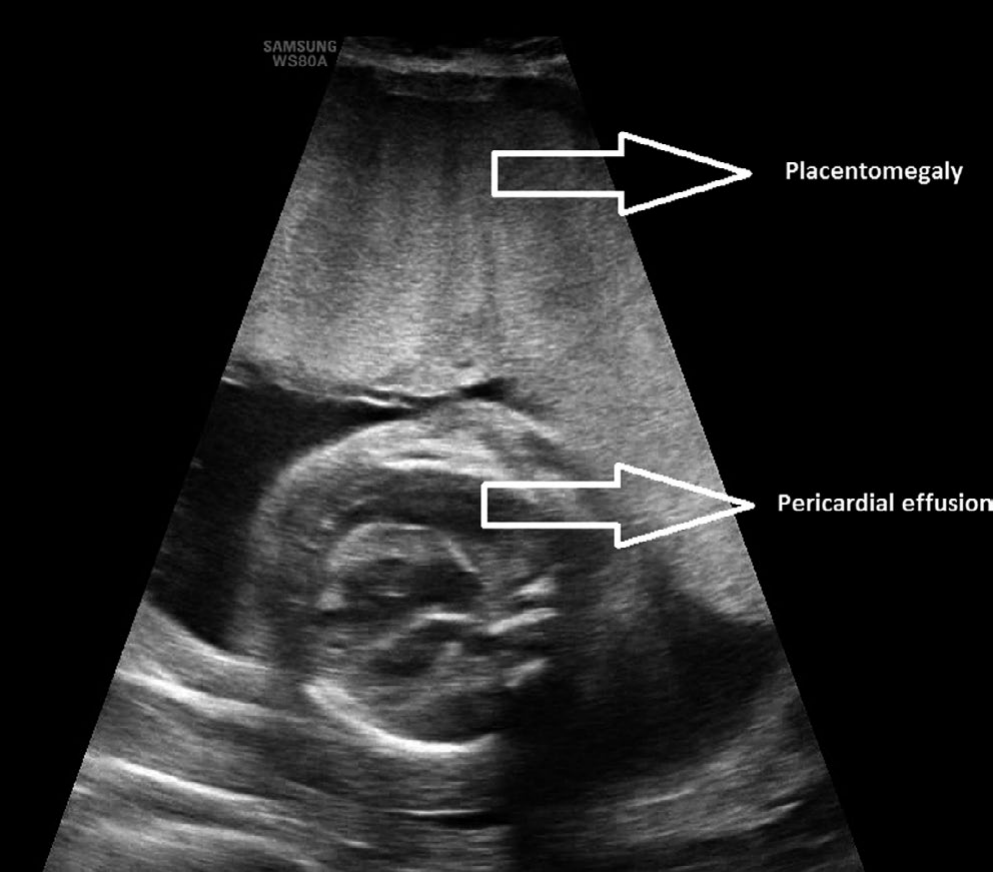
Fetal pericardial effusion and placentomegaly

**FIGURE 4:**
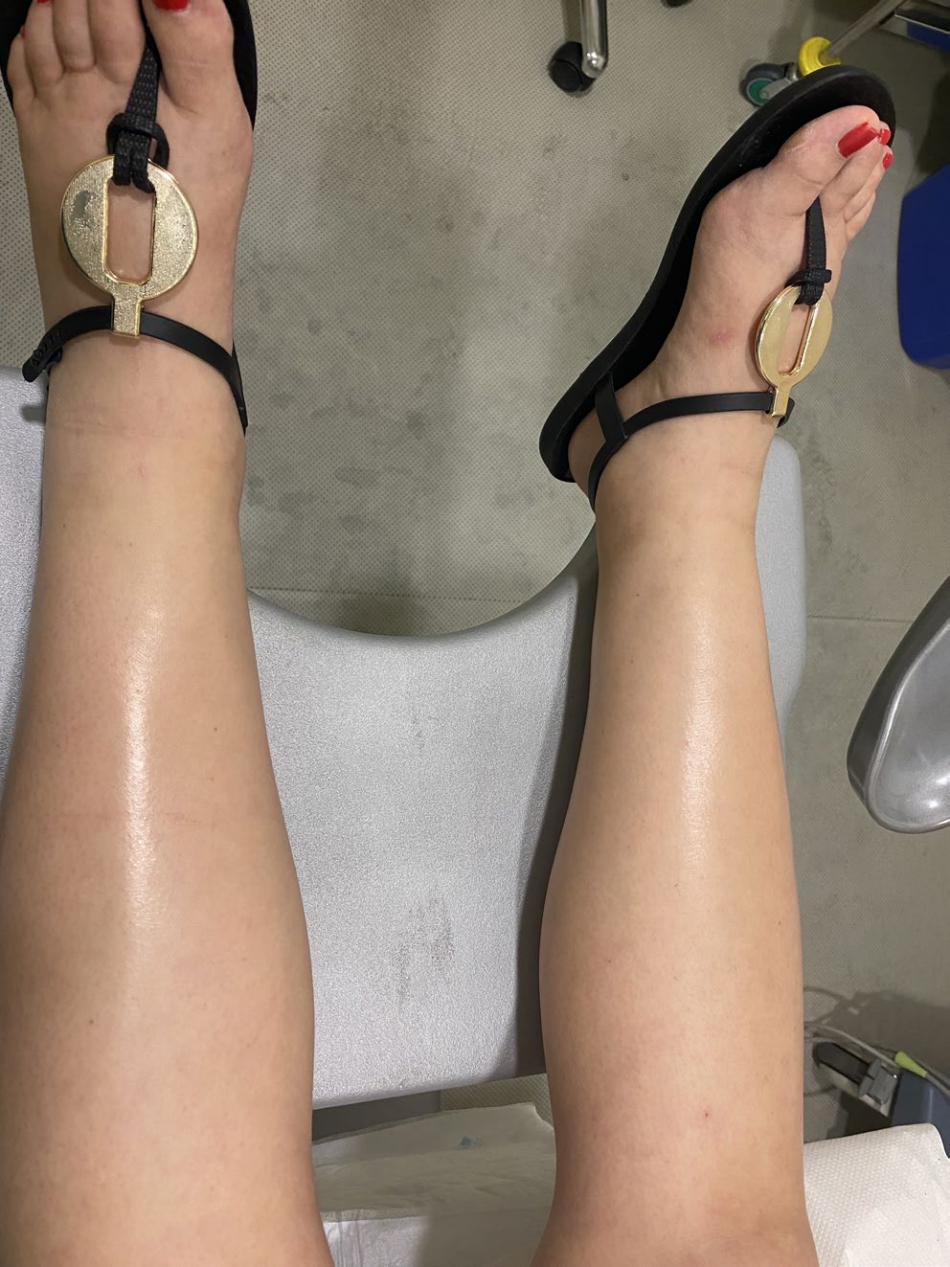
Maternal edema

The most relevant ultrasound images and clinical examination results for the patient are presented in Figures 1, 2, 3, 4.

## 4 OUTCOME AND FOLLOW‐UP

After 1 day of admission, the patient began to have regular and painful contractions with cervical dilation until she was diagnosed with spontaneous frank delivery. An emergency cesarean section was indicated for established labor, fetal malposition, and prematurity.

The perinatal results include the birth of a female neonate weighing 1000 g with evident signs of central predominant hydrops with marked ascites and placentomegaly. The umbilical arterial pH at birth was 7.31, and the Apgar test results were 2/2/2. The neonate required intubation and vasoactive drugs for stabilization. Immediately thereafter, she was admitted to the neonatal intensive care unit (NICU) that was necessary, where a blood test was performed that revealed severe neonatal anemia with hemoglobin of 4.2 g/dl and hematocrit of 14.6%. The neonate received transfusion with packed red blood cells and ascites drainage with no clinical improvement.

In the pretransfusion tests of the neonate, direct Coomb's test was positive (4+), and antibody screening with a 3‐cell panel was negative. Elution to identify the antibodies could not be performed due to the lack of a sample.

Given the suspicion of undiagnosed isoimmunization, an expanded panel study of irregular antibodies in the maternal blood was requested. Isoimmunization with anti‐Kpa was identified, with a titer of 1/16. Subsequently, the erythrocyte phenotype Kpa (+) was confirmed in the neonate, with maternal Kpa (−), Kpb (+), paternal Kpa (+), and Kbp (+).

Due to hemodynamic instability associated with prematurity and severe anemia, the newborn died at 19 h of age.

The patient continued to present mild hypertension during the immediate puerperium, which required control with descending doses of enalapril until it ceased. During the 7 days after delivery, the patient achieved a negative fluid balance, with an evident decrease in edema and normalization of laboratory values and proteinuria.

## 5 DISCUSSION

In this study, the authors present the case of a pregnant woman who met all of the diagnostic criteria for Mirror syndrome or Ballantyne syndrome. This syndrome is associated with intrauterine fetal death in 35.7% of cases; therefore, it is especially important to identify its cause to reduce possible adverse gestational events. The usual reasons for consultation in affected patients are maternal edema (up to 89.3%), increased blood pressure (60.7%), and headache and visual disturbances (14.3%). In analytical studies, mild anemia with hemodilution is a characteristic sign, in contrast to anemia and hemoconcentration, which can be present in other entities, such as preeclampsia. Proteinuria, elevated liver enzymes, and uric acid are also frequent.^5^, ^11^ Our patient presented the vast majority of the signs and symptoms previously described in the literature and associated findings in laboratory tests.

Regarding the precipitating causes of this condition, a systematic review conducted by Braun et al.^5^ in 2010 analyzed 56 cases compatible with this syndrome. A total of 28.6% of the cases were associated with fetal‐maternal isoimmunization, 17.9% with multiple pregnancies, 6.1% with viral infections, and the remaining 37.5% with fetal malformations, arrhythmias, and fetal or placental tumors. In the cases described in the literature, the symptoms disappeared on an average of 8.9 days after the pregnancy ended.^5^ In the particular case of our patient, the cause of the syndrome was isoimmunization resulting in fetal hydrops due to severe fetal anemia, and the patient's symptoms reversed 7 days after the end of pregnancy.

At present, less than 10% of cases of hydrops fetalis have an immune cause. The origin of its pathophysiology is the presence of maternal antibodies against fetal erythrocyte surface antigens. This isoimmunization usually originates during pregnancy or in cases of previous transfusions.^10^, ^12^ Our patient did not report previous transfusions but had a previous pregnancy that progressed normally.

In our environment, gestational screening for irregular antibodies is usually performed using an indirect Coomb's test. The panels used in maternal screening studies are capable of detecting the majority of clinically significant isoimmunizations^13^; however, they rarely include very low‐frequency erythrocyte antigens, such as Kpa, which are present in less than 2% of the Caucasian population and are even less common in other ethnic groups. This is why our patient was not identified with a high‐risk pregnancy by the gestational screening tests for isoimmunization.

Regarding isoimmunization with causes other than RhD, those involving other Rh group antigens (C, c, E, e) stand out for their frequency and clinical importance. Isoimmunization against Kell group antigens, including the Kpa antigen, is also clinically significant in pregnancy. The anti‐K antibody is particularly important because it can lead to neonatal hemolytic disease with enormous clinical severity. The anti‐Kpa antibody can be difficult to identify with routine screening tests, as occurred in our patient.^12^

In a high proportion of cases, fetal anemia can be diagnosed by measuring the maximum systolic peak of the middle cerebral artery (MCA). According to a systematic review published in 2019 that included 12 studies and 696 fetuses, MCA measurement presented a diagnostic sensitivity of 86%, with a specificity of 71% for predicting moderate‐severe fetal anemia.^14^ The anemia that develops in fetuses affected by antibodies to erythrocyte antigens is mainly caused by the suppression of erythropoiesis and, to a lesser extent, the hemolysis of fetal erythrocytes. For this reason, some articles suggest that fetal assessment by ultrasound measurement of the MCA may not be a good predictor of fetal anemia. This decreased diagnostic sensitivity in cases of isoimmunization could explain the presence of moderate and severe fetal anemia with normal MCA values, as occurred in our case.^15^

Within the Kell system, there are also antibodies that rarely cause erythroblastosis fetalis. Among them is anti‐Kpa. In the literature, there are only seven previous cases of perinatal involvement and three cases of fetal hydrops (Table 1). We, therefore, present the first case of mirror syndrome with fetal hydrops caused by anti‐Kpa to be described in the literature.

**TABLE 1:** Literature review

Author	Time of Alloimmunization diagnosis	Fetal symptoms	Maternal symptoms	Intrauterine treatment	Neonatal symptoms	Neonatal Treatment	Neonatal outcome
Smoleniec et al. 1994 ^17^	25 weeks	Hydrops fetalis	None	Intrauterine exchange transfusion	Neonatal anemia	RBC transfusion	Healthy
Koshy et al. 2013 ^18^	25 weeks	Hydrops fetalis	None	Intrauterine exchange transfusion	Neonatal anemia	RBC transfusion	Healthy
Le Vaillant et al. 2015 ^19^	27 weeks	Hydrops fetalis	None	Intrauterine exchange transfusion	Neonatal anemia	RBC transfusion	Healthy
Braumbaugh et al. 2011 ^20^	Postnatal	Hydrops fetalis	Hypertension	None	Neonatal anemia, purpura thrombocytopenia, coagulopathy, and hypoglycemia	Neonatal intensive care (RBC, platelet, plasma, and cryoprecipitate transfusion)	Healthy
Geczy et al. 1964 ^21^	Postnatal	Unknown	None	None	Neonatal anemia	RBC transfusion	Healthy
Costamagna et al. 1997 ^22^	Postnatal	None	None	None	Neonatal anemia	RBC transfusion and phototherapy	Healthy
Tuson et al. 2011 ^23^	Postnatal	None	None	None	Neonatal anemia	RBC transfusion	Healthy

Abbreviation: RBC, red blood cells.

Finally, regarding the perinatal management of Mirror syndrome, termination of pregnancy if the maternal condition worsens, since 21.4% of cases develop serious maternal complications, such as acute lung edema. In some reported cases of immune hydrops associated with this condition, intrauterine red blood cell transfusion has been performed, and maternal symptoms have disappeared after the correction of fetal anemia.^11^, ^16^

## AUTHOR CONTRIBUTIONS

JPM wrote the final manuscript, RAR reviewed the final manuscript, APC reviewed the final manuscript, VOA reviewed the final manuscript, and SGTL wrote the final manuscript.

## ETHICAL APPROVAL

This manuscript is the author's own original work, which has not been previously published elsewhere.

## CONSENT

Written informed consent was obtained from the patient to publish this report in accordance with the journal's patient consent policy.

## Data Availability

The data that support the findings of this study are available from the corresponding author upon reasonable request.
